# The predictive value of peripheral blood cell mitochondrial gene expression in identifying the prognosis in pediatric sepsis at preschool age

**DOI:** 10.3389/fcimb.2024.1413103

**Published:** 2024-07-24

**Authors:** Siyuan Jing, Yue Zhang, Wanling Zhao, Yifei Li, Yan Wen

**Affiliations:** ^1^ Key Laboratory of Birth Defects and Related Diseases of Women and Children of MOE, Department of Pediatrics, West China Second University Hospital, Sichuan University, Chengdu, Sichuan, China; ^2^ Department of Emergency, Chengdu Women’s and Children’s Central Hospital, School of Medicine, University of Electronic Science and Technology of China, Chengdu, Sichuan, China

**Keywords:** sepsis, mitochondrial DNA copy number, MT-CO1, Mt-ND1, MT-ATP6, prognosis

## Abstract

**Background:**

Sepsis represents a severe manifestation of infection often accompanied by metabolic disorders and mitochondrial dysfunction. Notably, mitochondrial DNA copy number (mtDNA-CN) and the expression of specific mitochondrial genes have emerged as sensitive indicators of mitochondrial function. To investigate the utility of mitochondrial gene expression in peripheral blood cells for distinguishing severe infections and predicting associated outcomes, we conducted a prospective cohort study.

**Methods:**

We established a prospective cohort comprising 74 patients with non-sepsis pneumonia and 67 cases of sepsis induced by respiratory infections, aging from 2 to 6 years old. We documented corresponding clinical data and laboratory information and collected blood samples upon initial hospital admission. Peripheral blood cells were promptly isolated, and both total DNA and RNA were extracted. We utilized absolute quantification PCR to assess mtDNA-CN, as well as the expression levels of mt-CO1, mt-ND1, and mt-ATP6. Subsequently, we extended these comparisons to include survivors and non-survivors among patients with sepsis using univariate and multivariate analyses. Receiver operating characteristic (ROC) curves were constructed to assess the diagnostic potential.

**Results:**

The mtDNA-CN in peripheral blood cells was significantly lower in the sepsis group. Univariate analysis revealed a significant reduction in the expression of mt-CO1, mt-ND1, and mt-ATP6 in patients with sepsis. However, multivariate analysis did not support the use of mitochondrial function in peripheral blood cells for sepsis diagnosis. In the comparison between pediatric sepsis survivors and non-survivors, univariate analysis indicated a substantial reduction in the expression of mt-CO1, mt-ND1, and mt-ATP6 among non-survivors. Notably, total bilirubin (TB), mt-CO1, mt-ND1, and mt-ATP6 levels were identified as independent risk factors for sepsis-induced mortality. ROC curves were then established for these independent risk factors, revealing areas under the curve (AUCs) of 0.753 for TB (95% CI 0.596–0.910), 0.870 for mt-CO1 (95% CI 0.775–0.965), 0.987 for mt-ND1 (95% CI 0.964–1.000), and 0.877 for mt-ATP6 (95% CI 0.793–0.962).

**Conclusion:**

MtDNA-CN and mitochondrial gene expression are closely linked to the severity and clinical outcomes of infectious diseases. Severe infections lead to impaired mitochondrial function in peripheral blood cells. Notably, when compared to other laboratory parameters, the expression levels of mt-CO1, mt-ND1, and mt-ATP6 demonstrate promising potential for assessing the prognosis of pediatric sepsis.

## Introduction

Currently, infectious diseases remain a predominant threat to children’s health. Alarming statistics reveal that over 13 million children worldwide succumb to infectious diseases annually, constituting 63% of child fatalities (Plunkett and Tong, 2015). Among these, sepsis, a rapidly advancing condition triggered by severe infection, not only stands as the leading cause of hospitalization and mortality in pediatric intensive care units (PICUs) but also represents a significant source of childhood disability. Consequently, a compelling clinical imperative exists to actively identify biomarkers characterized by high specificity and sensitivity for predicting the prognosis of patients with pediatric sepsis. Research has unearthed several candidates, such as serum-soluble urokinase-type plasminogen activator receptor (SuPAR), neutrophil CD64, pro-adrenomedullin, and cell-free plasma DNA (cfDNA), which exhibit the potential to forecast outcomes sepsis patient. However, out of more than 5,000 studies scrutinizing sepsis biomarkers from 2009 to 2019, merely 189 biomarkers linked to sepsis prognosis have been identified. Among these, only pro-adrenomedullin demonstrated a notably high predictive value, with an area under the curve (AUC) exceeding 0.8 (Pierrakos et al., 2020). Consequently, the quest for effective biomarkers to evaluate the prognosis of patients with sepsis remains ongoing. Therefore, it is critical to demonstrate a more efficient sepsis biomarker and assess its prognosis.

Mitochondria are not only the primary cellular energy source but also pivotal contributors to the immune response. They actively engage in the antiviral immune response through Toll-like and RIG-1-like receptor signaling pathways. They participate in the immune response against bacterial infections by regulating the production of reactive oxygen species (ROS) and orchestrating metabolic adaptations in phagocytic cells. Moreover, they play a critical role in facilitating the metabolic changes essential for differentiating T and B lymphocytes, significantly contributing to the adaptive immune response (Koch et al., 2017). It is imperative to note that mitochondrial dysfunction can impair immune cell functionality, and this immune dysfunction is intricately linked to the onset and progression of sepsis. The mitochondrial respiratory chain is the primary executor of mitochondrial function, comprising complexes I to IV. Its principal role involves the conveyance of hydrogen and electrons, with the energy generated in this process harnessed by complex V for the synthesis of ATP, which serves as the body’s energy currency. Within this intricate system, the mitochondrial genes mt-ND1, mt-CO1, and mt-ATP6 function as subunits of complex I, complex IV, and complex V, respectively. Both mitochondrial DNA copy number (mtDNA-CN) and mitochondrial gene expression serve as valuable indicators of mitochondrial function. Moreover, in the previous studies of our group, mtDNA-CN and specific mitochondrial genes expressions were associated with mitochondrial function, and several studies applied such parameters into mitochondrial functional assessment (Zhang et al., 2018; Yue et al., 2022). According to previous studies, mtDNA-CN was associated with several types of diseases’ prognosis via the critical regulation mechanisms of mitophagy in mitochondrial quality control (Liu et al., 2024). However, the specific genes’ expressions of mtDNA directly reflected the function of respiratory chain. Thus, assessing the exact expression of particular genes, such as mt-ND1, mt-CO1, and mt-ATP6, might serve as a more precious role in determining related clinical outcomes of infection. Moreover, in clinical practice, it was difficult to apply rapid ATP production measurement, as the mitochondria might be damaged during *ex vivo* transportation and storage. Thus, the examination of mtDNA-CN and specific genes expression would be much easier in clinical application, while numerous studies have explored the correlation between mtDNA-CN, mitochondrial gene expression, and infection.

Herein, we carried out this research to illustrate the changes of mtDNA-CN and mitochondrial gene expression (mt-ND1, mt-CO1, and mt-ATP6) in peripheral blood cells. Then, we attempted to identify the potential role of mtDNA-CN and mitochondrial gene expression in measuring the severity of infectious diseases and in predicting the prognosis of some patients with pediatric sepsis. The research revealed the advantages of mitochondrial DNA transcriptional levels in septic management compared with general laboratory parameters.

## Materials and methods

### Ethics statement

This study was conducted following the ethical guidelines and was approved by the Ethics Committee of West China Second University Hospital of Sichuan University (approval number: 2021–069). Informed consent was obtained from all parents or legal guardians of the patients, who provided their consent for including their child’s clinical and imaging details in the manuscript for publication purposes. This is a prospectively designed research. All participants in this study were hospitalized in either the respiratory department or the general intensive care unit of West China Second University Hospital of Sichuan University between January 2022 and December 2022. There were two groups involved in the observational cohort: the non-sepsis respiratory infection (non-S) group and the respiratory infection-induced sepsis (S) group.

### Inclusion and exclusion criteria

The inclusion criteria included the following (1): the diagnosis of sepsis meeting the criteria from the 2016 International Guidelines for Sepsis and Septic Shock, which should be induced by or have originated from initial respiratory infection (sepsis 3.0 diagnosis criteria); (2) the non-sepsis respiratory infection was recognized as bronchial pneumonia without other complications based on the Guidelines for the Management of Community-Acquired Pneumonia in Children in China; (3) the age of enrolled patients ranged from 2 to 6 years; and (4) the peripheral blood samples should be collected at the time of initial hospital admission before antibiotic agent supplementation.

The exclusion criteria were as follows: (1) patients with viral infection-associated sepsis; (2) patients with birth defects; (3) bronchopulmonary dysplasia history; (4) chest trauma history; (5) patients with immunodeficiency; (6) diagnosed with inherited metabolic disorders; (7) genetic test confirmed mitochondrial diseases; (8) patients with neoplastic diseases; (9) recent surgery history; (10) beyond respiratory or combined other system infections; (11) incomplete medical archive or laboratory tests; (12) lost to follow-up; and (13) severe chronic diseases.

The observational end point of the cohort had been designed as short-term follow-up of 1 month after hospital discharge or patient death. The clinical survival was considered as primary outcome.

### Blood sample collection and nucleotide extraction

Peripheral blood samples (4 mL) were collected from the patients on admission, of which 2 mL was stored in EDTA tubes and 2 mL was stored in RNA tubes; then, such samples were placed in a −40°C refrigerator. The experimental samples were kept and registered by special personnel, and the extraction time of RNA and DNA shall not exceed 6 months. Blood DNA Kit (D3392–02) and Blood RNA Kit (R6814–02, Omega, USA) were used to extract DNA and RNA from peripheral blood samples, and the concentration and purity of DNA and RNA were determined and stored at −20°C.

### Plasmid construction and mtDNA-CN measurement

To obtain the DNA sequence amplification of mt-ND1, design primers (Forward—ATACCCATGGCCAACCTCCTA; Reverse—TAGGTTTGAGGGGGAATGCTG) were used to amplify the whole mitochondrial mt-ND1 gene to quantify the mitochondrial genome. Fragments were amplified using NEB Q5 High-Fidelity DNA polymerase. Then, the mt-ND1 sequence was connected to the T vector according to the Mighty TA-cloning Reagent Set for Prime STAR^®^ (Code N0.6019 TaKaRa Company) kit instructions for research and the plasmid was linearized to make a gradient concentration standard. The number of DNA copies was calculated according to the following formula:

DNA (copies/μL) = (DNA concentration (ng/μL) × 6.022 × 10^23^)/(Length (bp) × 1 × 109 × 660) [6.022 × 10^23^ = Avogadro’s constant, 1 × 10^9^ = mole amount converted to ng, 660 = average mass of a pair of double-stranded DNA bases (g), Length = vector + length of insert]. Dilute the linearized plasmid 10-fold with ddH_2_O to a concentration of 10^3^–10^8^ copies/μL to make a standard.

### RT-qPCR quantification of MT-CO1, MT-ND1, and MT-ATP6

RNA was reverse-transcribed into complementary deoxyribonucleic acid [complementary DNA (cDNA)] (according to HiScriptIII AII-in-one RT SuperMix Perfect for qPCR), and then RT-PCR reaction was performed (Qiagen SYBR Green Mixture reagent protocol). The relative gene expression was calculated using the 2^−ΔΔCt^ method. The sequence of each gene was amplified, as shown in **Table 1**.

**Table 1:** Gene amplification primer sequence.

Gene	Primer sequence (5′-3′)
*MT-CO1 Forward*	TCTCAGGCTACACCCTAGACCA
*MT-CO1 Reverse*	ATCGGGGTAGTCCGAGTAACGT
*MT-ND1 Forward*	GGCTATATACAACTACGCAAAGGC
*MT-ND1 Reverse*	GGTAGATGTGGCGGGTTTTAGG
*ATP6 Forward*	GAAGCGCCACCCTAGCAATA
*ATP6 Reverse*	GCTTGGATTAAGGCGACAGC
*18S Forward*	TTGACGGAAGGGCACCACCAG
*18S Reverse*	GCACCACCACCCACGGAATCG

### Statistical analysis

Statistical analysis was performed by GraphPad Prism software (Version 8, San Diego, CA). Normality test would be completed initially, and the data were presented as mean ± SD once they passed normality test. Otherwise, the continuous data would be presented in median with range, while the categorical variables were expressed as *n* (%). For continuous variable data passed normality test, an independent-sample *t*-test was used between two groups; for categorical variables, a chi-square test was used for comparison. When *p* < 0.05, it was judged as a significant statistical difference. Univariate and multivariate analyses had been applied to analyze the clinical and laboratory indicators and the expression of mt-CO1, mt-ND1, and mt-ATP6 between survivors and non-survivors in sepsis, and identify particular independent risk factors. Then, SPSS 22.0 software had been used to illustrate the ROC curve of independent risk factors to predict adverse clinical outcome of sepsis, and AUC had been used to clarify the test efficiency.

## Results

### Characters of included patients

Ninety-three patients were initially enrolled in the non-S group. Seven patients were subsequently excluded due to lost follow-ups, while three patients were identified as having congenital disorders, seven had concomitant injuries to other systems, and two patients did not complete coagulation tests. Thus, the final analysis included 74 patients in the non-S group. In the S group, 97 patients were initially included. Among them, 8 patients had congenital birth defects, 5 patients had positive genetic analysis results, 3 were diagnosed with immunodeficiency diseases, 2 were previously diagnosed with bronchopulmonary dysplasia, and 12 patients had concurrent COVID-19 either before or after the sepsis diagnosis. Consequently, 67 cases with complete medical records were included for further analysis. Baseline information was compared between the two groups, revealing no significant differences in gender distribution, average age, or body weight. There were 51 patients (76%) in the S group identified with positive results by blood culture or metagenomics analysis, including 17 cases with *S. aureus*, 15 cases with *S. pneumoniae*, 7 cases with *P. aeruginosa*, 5 cases with *H. influenzae*, 3 cases with *K. pneumoniae*, 3 cases with *E. coli*, and 1 case with *E. faecium*. However, the S group had a substantially longer average hospital stay duration (23.68 ± 7.74 days) than the non-S group (4.74 ± 1.37 days). Additionally, the S group experienced 11 in-hospital non-survivors, whereas no deaths were recorded in the non-S group (**Table 2**).

**Table 2:** Univariate analysis of laboratory tests and mitochondrial function between non-S and S groups.

Variable	Non-S (*n* = 74)	S (*n* = 67)	*p*
Female/male (*n*)	27/47	36/31	
Age (m)	45.5 ± 14.23	40.25 ± 17.60	
Weight (kg)	16.24 ± 3.31	15.39 ± 4.48	
Hospital duration (days)	4.74 ± 1.37	23.68 ± 7.74	<0.001
Death (*n*)	0 (0%)	5 (7.46%)	0.022
Blood culture or metagenomics analysis	Not involved	51 (76%) *S. aureus* (17/25.4%) *S. pneumoniae* (15/22.4%) *P. aeruginosa* (7/10.4%) *H. influenzae* (5/7.5%) *K. pneumoniae* (3/4.5%) *E. coli* (3/5.4%) *E. faecium* (1/1.5%)	
WBC (10^9^/L)	15.77 ± 22.46	11.54 ± 7.81	0.145
N (%)	62.33 ± 17.79	58.06 ± 24.31	0.232
L (%)	27.34 ± 17.06	29.61 ± 19.47	0.461
HGB (g/L)	119.05 ± 9.04	101.13 ± 27.60	<0.001
CRP (mg/L)	35.21 ± 30.99	72.45 ± 69.06	<0.001
PCT (μg/L)	1.18 ± 1.04	12.59 ± 27.31	<0.001
ALT (U/L)	21.97 ± 9.38	192.72 ± 436.93	<0.001
AST (U/L)	42.76 ± 10.47	301.22 ± 754.10	<0.001
TB (mmol/L)	5.24 ± 2.51	16.95 ± 25.92	<0.001
ALB (g/L)	42.83 ± 2.81	39.20 ± 22.34	0.167
GLB (g/L)	22.48 ± 3.44	19.65 ± 7.34	0.003
γ-GT (U/L)	9.98 ± 3.62	62.07 ± 96.96	<0.001
LDH (U/L)	342.37 ± 90.97	1943.11 ± 9117.90	0.133
UN (mmol/L)	7.04 ± 26.39	6.77 ± 9.04	0.938
Cr (μmol/L)	21.94 ± 4.83	64.07 ± 112.43	0.001
PT (s)	13.28 ± 0.65	16.12 ± 16.95	0.150
APTT (s)	30.28 ± 2.94	35.21 ± 11.79	<0.001
Fg (g/L)	4.05 ± 0.54	304.72 ± 171.36	<0.001
INR	1.44 ± 1.11	1.32 ± 0.35	0.385
DDI (μg/mL)	1.04 ± 0.25	7.39 ± 10.44	<0.001
FDP (μg/mL)	3.05 ± 0.59	31.78 ± 80.25	0.002
2-ΔΔCT-CO1	60.89 ± 128.21	1.41 ± 0.96	<0.001
2-ΔΔCT-ND1	101.90 ± 210.26	1.58 ± 0.98	<0.001
2-ΔΔCT-ATP6	64.90 ± 132.77	1.33 ± 0.90	<0.001

Non-S, non-sepsis respiratory infection; S, respiratory infection-induced sepsis. Numerical variables were expressed as mean ± SD; categorical variables were presented as percentage.

### Severe infection induced reduction of mtDNA-CN quantification

Both the plasmid standard product with a concentration of 10^3^–10^8^ copies/μL and the DNA samples from the two groups were simultaneously subjected to fluorescent qPCR. Following the completion of multiple batches, a standard curve was constructed. The quantitative templates with various concentrations of standards exhibited a linear correlation with the Ct value. The regression equations for the mt-ND1 standard curve were derived as follows: *y* = −4.146*x* + 39.575, where *y* represents Ct, *x* signifies the base 10 logarithm of the plasmid template’s copy number, and the correlation coefficients reached 0.9939. Subsequently, the Ct value was substituted into the regression equation of the standard curve to calculate the absolute quantity of mtDNA-CN. Based on the Ct values obtained from qPCR assessment, the average mtDNA-CN was 10^5.95 ± 0.32^ in the S group and 10^6.11 ± 0.15^ in the non-S group. These findings highlight a significant decrease in mtDNA-CN among patients with sepsis. Consequently, it becomes crucial to assess the expression of specific mitochondrial genes transcription from mtDNA and analyze their predictive value in early identification of adverse prognosis associated with sepsis.

### Mitochondrial DNA expression level failed to serve as an independent factor for severe infection

The clinical characteristics of patients in the non-S group and S group are presented in **Table 2**. Univariate analysis had been used to analyze the clinical and laboratory parameters between the two groups. It was found that the values of HBG, CRP, PCT, ALT, AST, TB, GLB, γ-GT, Cr, APTT, Fg, DDI, FDP, 2-ΔΔCT-CO1, 2-ΔΔCT-ND1, and 2-ΔΔCT-ATP6 were significantly different between two groups (*p* < 0.05), as shown in **Table 2**. Then, logistic regression had been used for multivariate analysis to evaluate whether such parameters would contribute as independent factors in distinguishing severe conditions among patients with respiratory infection. Among the included variables that were identified based on univariate analysis, HGB (OR = 0.78, *p* = 0.031), TB (OR = 1.447, *p* = 0.003), γ-GT (OR = 2.531, *p* = 0.009), Cr (OR = 1.392, *p* = 0.034), and FDP (OR = 2.647, *p* = 0.002) demonstrated their potential in serving as independent risk factors for distinguishing severe respiratory infection, although the expression of mt-CO1, mt-ND1, and mt-ATP6 had been recorded to be extremely reduced in the S group. However, the logistic regression analysis of the values among 2-ΔΔCT-CO1, 2-ΔΔCT-ND1, and 2-ΔΔCT-ATP6 presented no significant meaning in separating sepsis and non-severe infection (**Table 3**). Therefore, although the expression of mitochondrial DNA coding gene is significantly downregulated in patients with sepsis, the expression of mitochondrial DNA coding genes failed to serve as an independent factor to distinguish sepsis.

**Table 3:** Multivariate analysis to identify independent risk factors between Non-S and S groups.

Variable	*B*	S.E.	Wald	OR	*p*
HGB	−0.248	0.115	4.634	0.780	0.031
CRP	0.032	0.018	3.024	1.033	0.082
PCT	1.505	0.800	3.537	4.504	0.060
ALT	−0.017	0.055	0.090	0.983	0.764
AST	0.074	0.079	0.876	1.077	0.349
TB	0.370	0.126	8.563	1.447	0.003
GLB	−0.317	0.194	2.674	0.728	0.102
GT	0.929	0.355	6.841	2.531	0.009
Cr	0.331	0.156	4.490	1.392	0.034
APTT	0.032	0.041	0.599	1.032	0.439
Fg	0.447	26.787	0.000	1.564	0.987
DDI	−0.096	0.202	0.223	0.909	0.637
FDP	0.974	0.319	9.340	2.647	0.002
2-ΔΔCT-CO1	0.462	0.796	0.337	1.587	0.561
2-ΔΔCT-ND1	−0.651	0.697	0.873	0.521	0.350
2-ΔΔCT-ATP6	0.206	0.337	0.373	1.229	0.541

### Downregulated mitochondrial gene transcription was associated with septic lethality

In the next step analysis, we also performed univariate analysis to demonstrate potential factors in predicting adverse clinical outcomes among patients with sepsis. According to the initial results, the values of TB, UN, PT, APTT, FDP, 2-ΔΔCT-CO1, 2-ΔΔCT-ND1, and 2-ΔΔCT-ATP6 presented significant differences between the septic survivors and non-survivors (*p* < 0.05, **Table 4**). Then, logistic regression has been used to evaluate the potential role of the initially identified parameters that serve as an independent factor to predict septic lethality (**Table 5**). According to the results, the expressions of TB (OR = 1.042, *p* = 0.037), mt-ND1 (OR = 0.00, *p* = 0.021), mt-CO1 (OR = 0,004, *p* = 0.004), and mt-ATP6 (OR = 0.007, *p* = 0.008) revealed persistent strength in assessing clinical outcomes of sepsis, which could serve as independent factors in early identifying non-survivors.

**Table 4:** Univariate analysis between survivors and non-survivors in patients with sepsis.

Variable	Survivors (*n* = 56)	Non-survivors (*n* = 11)	*p*
WBC (10^9^/L)	11.74 ± 13.78	10.53 ± 6.20	0.642
N (%)	56.62 ± 44.69	65.84 ± 24.58	0.243
L (%)	30.27 ± 32.39	26.65 ± 24.64	0.575
N/L (%)	4.04 ± 12.25	6.03 ± 6.05	0.352
HGB (g/L)	99.98 ± 59.18	107 ± 25.89	0.445
CRP (mg/L)	80.33 ± 131.65	35.18 ± 59.08	0.055
PCT (μg/L)	12.05 ± 65.07	14.17 ± 33.89	0.827
ALT (U/L)	172.12 ± 948.46	295.72 ± 327.50	0.395
AST (U/L)	286.81 ± 1638.05	373.27 ± 422.36	0.731
TB (mmol/L)	12.31 ± 22.57	22.4 ± 15.31	0.025
ALB (g/L)	35.09 ± 11.43	32.85 ± 6.85	0.268
GLB (g/L)	19.95 ± 17.03	18.13 ± 6.27	0.456
γ-GT (U/L)	63.32 ± 259.50	55.72 ± 25.01	0.814
LDH (U/L)	2040.5 ± 27463.45	1447.36 ± 1931.94	0.845
UN (mmol/L)	5.34 ± 18.25	11.31 ± 7.75	0.013
Cr (μmol/L)	51.35 ± 229.23	101.54 ± 104.98	0.106
PT (s)	13.77 ± 6.35	29.08 ± 40.48	0.005
APTT (s)	33.97 ± 17.63	42.02 ± 18.57	0.038
Fg (g/L)	310.67 ± 367.22	272.3 ± 165.72	0.501
INR	1.30 ± 0.67	1.46 ± 0.42	0.191
DDI (μg/mL)	6.81 ± 15.91	10.59 ± 14.69	0.276
FDP (μg/mL)	21.23 ± 47.33	90.08 ± 182.02	0.008
2-ΔΔCT-CO1	1.56 ± 1.30	0.69 ± 0.24	0.005
2-ΔΔCT-ND1	1.79 ± 1.60	0.52 ± 0.13	0.000
2-ΔΔCT-ATP6	1.48 ± 1.55	0.54 ± 0.18	0.001

Numerical variables were expressed as mean ± SD; categorical variables were presented as percentage.

**Table 5:** Multivariate analysis reveals the independent risk factors in assessing non-survivors in sepsis.

Variable	*B*	S.E.	Wald	OR	*p*
TB	0.041	0.024	2.934	1.042	0.037
UN	0.073	0.047	2.393	1.075	0.122
PT	0.070	0.127	0.300	1.072	0.584
APTT	0.016	0.035	0.202	1.016	0.653
FDP	0.004	0.013	0.102	1.004	0.750
MT-CO1	−5.628	1.948	8.350	0.004	0.004
MT-ND1	−29.312	12.659	5.362	0.000	0.021
MT-ATP6	−4.896	1.841	7.076	0.007	0.008

**Figure 1:**
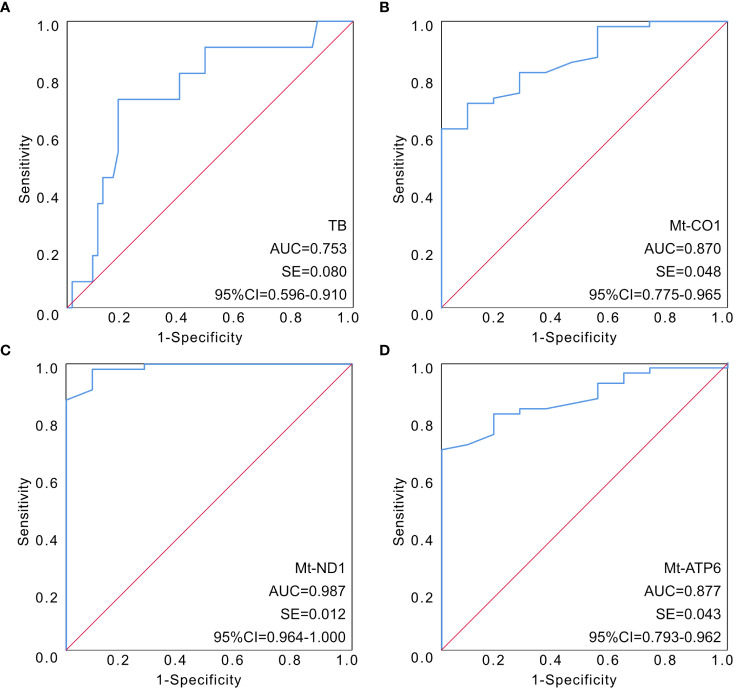
The established ROC curves of identified parameters according to multivariable analysis. **(A)** The AUC of the ROC curve based on TB was 0.753 (95% CI 0.596–0.910). **(B)** The AUC of the ROC curve based on mt-CO1 was 0.870 (95% CI 0.775–0.965). **(C)** The AUC of the ROC curve based on mt-ND1 was 0.987 (95% CI 0.964–1.000). **(D)** The AUC of the ROC curve based on mt-ATP6 was 0.877 (95% CI 0.793–0.962). AUC, area under the curve; ROC, receiver operating characteristic; TB, total bilirubin.

In order to further clarify the predictive value of mitochondrial DNA expression assessment in the clinical outcome of sepsis, ROC curves had been illustrated to determine the predictive value of different indicators. The AUC of the ROC among the four parameters had been calculated as 0.753 for TB (95% CI 0.596–0.910), 0.870 for mt-CO1 (95% CI 0.775–0.965), 0.987 for mt-ND1 (95% CI 0.964–1.000), and 0.877 for mt-ATP6 (95% CI 0.793–0.962) (**Figure 1**; **Table 6**).

**Table 6:** AUC of particular ROC curves in determining survivor in patients with sepsis.

Variable	AUC	S.E.	95% CI
TB	0.753	0.080	0.596–0.910
MT-CO1	0.870	0.048	0.775–0.965
MT-ND1	0.987	0.012	0.964–1.000
MT-ATP6	0.877	0.043	0.793–0.962

AUC, area under the curve; CI, confidence interval; S.E., standard error; TB, total bilirubin.

## Discussion

The pathogenesis of sepsis is inherently intricate. Traditionally, it has been attributed to the body’s response to infection becoming imbalanced. Upon entering the body, pathogens engage with pathogen-related molecular patterns, triggering immune cell activation and inflammatory responses. These responses, in turn, set off complex cascade reactions within and between cells, intensifying the inflammatory processes. This ultimately results in heightened capillary permeability and vasodilation, leading to decreased blood pressure, tissue underperfusion, and abnormal blood coagulation. Notably, research has demonstrated a reciprocal promotion between inflammatory responses and coagulation abnormalities in sepsis. Further investigations have revealed that the initial stages of sepsis are characterized by inflammation and immune effector cell activation aimed at pathogen clearance (Cheng et al., 2016; Weiss et al., 2020). As sepsis progresses, an anti-inflammatory response gains prominence, accompanied by diminished counts and compromised function of immune cells, including monocytes, lymphocytes, and dendritic cells. This phase also witnesses reduced expression and production of pro-inflammatory cytokines, coupled with increased anti-inflammatory factors, all working together to mitigate inflammation and facilitate tissue repair. Evidently, immune dysfunction plays a pervasive role throughout the onset and progression of sepsis, and the intertwined dynamics of inflammatory response and coagulation dysfunction closely contribute to its development.

Mitochondria possess their own genome, known as mt-DNA, which stands as the sole DNA molecule residing in the human cytoplasm independently from the nuclear chromosomes. The quantification of these genomes, termed mtDNA-CN, serves as a reflection of mitochondrial size, quantity, and functional status (Malik and Czajka, 2013). Mitochondria, dynamic organelles crucial for cellular homeostasis, serve as the primary site for ATP production. They also engage in fatty acid β-oxidation and the tricarboxylic acid cycle, regulate cellular calcium metabolism and signal transduction, and play a role in programmed cell death. Moreover, mitochondria actively contribute to the innate immune response during pathogen infections through various pathways. Concurrently, mitochondria are essential for the metabolic transformations required for the differentiation of T and B lymphocytes and play a pivotal role in the adaptive immune response (Koch et al., 2017). Research indicates that during sepsis, immune cells experience mitochondrial dysfunction, characterized by decreased mitochondrial membrane potential in peripheral blood mononuclear cells (PBMCs) and energy depletion (Japiassu et al., 2011; Weiss et al., 2015). Additionally, the activity of mitochondrial complexes is variably diminished (Brealey et al., 2002; Rosca et al., 2011; Garrabou et al., 2012; Singh et al., 2016). Dysfunctional mitochondria profoundly impact immune cell activity, ultimately leading to dysregulated immune responses.

Altered mitochondrial function has been linked to a spectrum of diseases, encompassing neoplastic diseases (Reznik et al., 2016), Parkinson’s disease (Pyle et al., 2016), cardiovascular diseases (Ashar et al., 2017; Yue et al., 2018), hepatitis C (Yue et al., 2018), acquired immunodeficiency syndrome (Quick et al., 2017), and novel coronavirus pneumonia (Shenoy, 2020). In the context of sepsis, Pyle et al. discovered that the blood cells of surviving patients with sepsis exhibited significantly higher mt-DNA levels compared to those in the non-surviving group. The increased mt-DNA levels were closely associated with the prognosis of patients with sepsis (Pyle et al., 2010). Another researcher reported the predictive value of plasma mt-DNA for the early diagnosis and prognosis in patients with sepsis (Yan et al., 2018; Yang et al., 2019). Busani et al. conducted a study on the expression of mt-DNA genes in peripheral blood and its association with clinical outcomes in patients with sepsis in the PICU. Their findings indicated that mt-DNA can serve as a biomarker for disease severity and even mortality in both adults and children receiving critical care (Busani et al., 2020). MT-CO1 is a functional subunit of cytochrome C oxidase (COX). Mutations in the mt-CO1 coding region lead to structural and functional changes in COX, resulting in increased production of ROS, apoptosis, and genetic susceptibility to sepsis. Moreover, the mt-DNA T6459C mutation is responsible for mitochondrial damage in the early stages of sepsis, leading to significant pathological alterations associated with sepsis (Shen et al., 2018). Rahmel et al. observed reduced abundance of mitochondrial TFAM in PBMCs of patients with sepsis. This reduction was accompanied by diminished mt-DNA copy number, decreased expression of MT-ND1, and a decline in cellular ATP content (Rahmel et al., 2020).

In our research, we detected the mtDNA-CN of peripheral blood cells between non-S and S groups. The results showed that the mt-DNA copy number of children in the severe infection group was significantly lower. It suggested that the mitochondrial function of blood cells had been impaired in severe infection. However, the expression of mt-CO1, mt-ND1, and mt-ATP6 failed to demonstrate any statistical significance and could not work as an independent factor for distinguishing severe infection. Furthermore, we identified that the expression of mt-ND1, mt-CO1, and mt-ATP6 was associated with sepsis-related death, which had been confirmed by AUC under ROC calculations. According to the results, the expression of mitochondrial genes presented a much higher predictive value than previous applied clinical parameters. Therefore, it was believed that mitochondrial function revealed an efficient predictive value in determining the adverse clinical outcome of children with sepsis.

To date, numerous biomarkers for predicting sepsis prognosis have been extensively investigated. Studies have revealed that elevated levels of CRP and PCT are indicative of poor outcomes in patients with sepsis. Specifically, CRP has demonstrated a favorable discriminative capacity for predicting mortality in children with sepsis beyond 6 months in the PICU. However, the calculated AUC of CRP was not satisfied enough, and other newly defined parameters were warranted (Cui et al., 2019; Tonial et al., 2021). Early studies also highlighted the prognostic potential of cfDNA in predicting sepsis mortality. Dwivedi et al. demonstrate that the AUC for predicting sepsis mortality of cfDNA is notably high at 0.97 (95% CI 0.93–1.00), presenting a perfect predictive value for septic lethality (Dwivedi et al., 2012). Subsequently, Forsblom et al. identified cf-DNA as an independent prognostic marker for death in patients with sepsis (Forsblom et al., 2014). However, more recent literature reports with a higher sample size have suggested that cfDNA, with an average AUC around 0.76, exhibited less favorable specificity and sensitivity in predicting death among patients with general sepsis (Rannikko et al., 2018). Moreover, other studies demonstrate that neutrophil CD64 expression shows a relatively weak association with sepsis mortality (AUC < 0.8) (Livaditi et al., 2006; Dimoula et al., 2014), while the AUCs of CYSTM1, MMP8, and CD177, which are strongly associated with a variety of immune cells, were 0.988, 0.973, and 0.986, respectively, in predicting pediatric sepsis (Zhang et al., 2023). Notably, several studies have emphasized the sensitivity and specificity of plasma-soluble urokinase-type plasminogen activator receptor (suPAR) as an independent prognostic biomarker in patients with sepsis (Huttunen et al., 2011; Koch et al., 2011; Larsen and Petersen, 2017). While it surpasses PCT and CRP in predicting patient mortality, its AUC remains relatively low, with correspondingly low specificity and sensitivity (Donadello et al., 2012; Donadello et al., 2014; Huang et al., 2020). In a recent meta-analysis focused on sepsis mortality, proadrenomedullin was the sole biomarker to achieve an AUC exceeding 0.8, accompanied by high specificity (92% specificity; 75% sensitivity) (Pierrakos et al., 2020). Moreover, in recent years, more and more studies have focused on the predictive value of different biomarker combinations for pediatric sepsis; the AUC of these combinations can even reach 1 (Li et al., 2023; Wang et al., 2023; Leonard et al., 2024). Remarkably, in our study, mitochondrial function-related indicators, including mt-CO1, mt-ND1, and mt-ATP6, exhibited significantly higher discriminative capabilities for predicting the clinical outcomes of sepsis-associated lethality compared to traditional laboratory indicators. Therefore, we contend that mitochondrial function indices hold substantial predictive value for the clinical outcomes of sepsis in children.

This study has several limitations that warrant consideration: The sample size in this study is relatively small, and it would benefit from validation through the expansion of the sample cohort. Meanwhile, the patients we included ranged in age from 2 to 6 years, which should be expanded to 0–18 years in future studies. However, the results from this prospective cohort study are deemed credible and hold significant scientific value due to the detailed statistical analyses conducted. The study assessed mitochondrial function by measuring mtDNA-CN and mitochondrial gene expression. It lacks direct methods to evaluate mitochondrial function, which should be addressed in subsequent investigations.

## Conclusion

The morbidity and mortality rates associated with pediatric sepsis continue to pose a significant challenge. Early prognosis prediction serves as a valuable tool for clinicians, aiding in the assessment of potential disease progression and informing therapeutic decisions. Both mtDNA-CN and the expression of mitochondrial genes have shown associations with the severity and clinical outcomes of infectious diseases. Moreover, severe infections have been observed to impair the mitochondrial function of peripheral blood cells. In comparison to other laboratory parameters, the expression levels of mt-CO1, mt-ND1, and mt-ATP6 demonstrate notable potential in predicting the prognosis of pediatric sepsis.

## Data availability statement

The original contributions presented in the study are included in the article/supplementary material. Further inquiries can be directed to the corresponding authors.

## Ethics statement

This study was approved by the Ethics Committee of West China Second University Hospital of Sichuan University (2021069). Informed consent from all the patient’s parents was obtained, including the patient’s clinical and imaging details in the manuscript for the purpose of publication.

## Author contributions

SJ: Conceptualization, Data curation, Investigation, Methodology, Resources, Validation, Writing – original draft. YZ: Data curation, Formal analysis, Investigation, Methodology, Writing – original draft. WZ: Investigation, Methodology, Resources, Software, Validation, Writing – original draft. YL: Conceptualization, Data curation, Formal analysis, Funding acquisition, Investigation, Methodology, Project administration, Software, Supervision, Validation, Writing – original draft, Writing – review & editing. YW: Conceptualization, Data curation, Investigation, Methodology, Project administration, Resources, Validation, Writing – review & editing.
